# Development and validation of the attitude towards Surrogacy Scale in a polish sample

**DOI:** 10.1186/s12884-023-05751-x

**Published:** 2023-06-03

**Authors:** Karolina Lutkiewicz, Łucja Bieleninik, Paweł Jurek, Mariola Bidzan

**Affiliations:** 1 Institute of Psychology, University of Gdansk, 80-309 Gdansk, Poland; 2 GAMUT-The Grieg Academy Music Therapy Research Centre, NORCE Norwegian Research Centre, Bergen, Norway

**Keywords:** Surrogacy, Attitude, Attitude Scale, Psychometrics, Validation

## Abstract

**Background:**

Surrogacy is one of the options in reproductive medicine that raises a lot of ethical, legal and psychological controversy. Surveying attitudes toward surrogacy plays an important role in building awareness of this phenomenon in the society, which may help eliminate its stigma. In this study authors aimed to develop and validate a scale to assess the attitudes towards surrogacy.

**Methods:**

In this study cross-sectional design was implemented. Development process of the Attitude towards Surrogacy Scale (ATSS) included items development based on literature reviews, other existing questionnaires, confirmatory factor analysis (CFA), and reliability analysis using internal consistence coefficients. A pilot study using adult members of the public was conducted after consultation with the Expert Advisory Panel Board. The final survey, which was used in this study, consisted of 24 items, which were organized into the four subscales: general opinion on surrogacy and its social context (7 items), financing and legalizing surrogacy (8 items), the acceptance of surrogacy (4 items), and attitudes towards the intended parents and children born through surrogacy (5 items). 442 individuals participated in this study.

**Results:**

The final version of Attitude towards Surrogacy Scale (ATSS) consists of 15 items, grouped in three subscales. Final version of the ATSS showed that the three-factor model indicated an acceptable model fit: Chi-square = 320.46, p < 0.01, df = 87, CFI = 0.94, TLI = 0.92, RMSEA = 0.078 (90% C.I.: 0.070–0.086), SRMR = 0.040 Reliability was assessed by calculating the McDonald's omega that ranged from 0.74 for the Surrogacy ethical context subscale to 0.94 for the overall ATSS score*.*

**Conclusion:**

ATSS was developed to measure general attitude toward surrogacy with satisfying psychometric properties. The analysis of socio-demographic variables with ATSS showed that the most significant predictor of the general attitude towards surrogacy, and three aspects of surrogacy was being a religious person (profess a Catholic religion or profess another religion).

**Supplementary Information:**

The online version contains supplementary material available at 10.1186/s12884-023-05751-x.

## Abstract (plain language summary)

Surrogacy—is one of the most controversial methods of infertility treatment. This concept is associated with difficult ethical, psychological and social issues, in which each aspect may lead to different trends in attitudes. The study of attitudes in society plays an important role in the analysis of various aspects of a given phenomenon, helps to fill legal gaps and ambiguities, and to transform controversial dimensions into normative concepts. Thus, this study aimed to develop and validate a scale to assess the attitudes towards surrogacy.

The survey used in this study, consisted of 24 questions, which were organized into the four subscales: general opinion on surrogacy and its social context (7 items), financing and legalizing surrogacy (8 items), the acceptance of surrogacy (4 items), and attitudes towards the intended parents and children born through surrogacy (5 items). 442 individuals participated in this study. The final version of Attitude towards Surrogacy Scale consists of 15 questions, grouped in three subscales: opinion on surrogacy ethical context, financing and legalizing surrogacy, and an acceptance of surrogacy.

This is the first scale in Poland for the assessment of general attitudes towards surrogacy. This measure allows to capture the opinions towards three aspects of surrogacy: surrogacy’s ethical context, the financing and legalizing surrogacy, and acceptance of surrogacy. The scale can be addressed to various groups in the society, not only for studies related to reproductive medicine.

**Supplementary Information:**

The online version contains supplementary material available at 10.1186/s12884-023-05751-x.

## Introduction

### Background

Surrogacy is a known phenomenon which dates to biblical times [1, 2]. Together with reproductive innovations and the introduction of the In vitro fertilization (IVF) method, where artificial insemination (the fertilization of an egg) can take place in a laboratory, surrogacy is one of the options in reproductive medicine to overcome infertility problems. It began to flourish in the USA in the 1980s, and today has grown worldwide into a global trend [2].

Surrogacy as a form of assisted reproduction [3], is an arrangement that involves using a third party, a ‘surrogate mother’ or ‘surrogate’. A surrogate mother carries and delivers a child on behalf of a couple, who will become the child's parents after birth. Nevertheless, it must be mentioned that definitions of the terms related to surrogacy are still not fully clear, especially from a legal perspective. In most countries, the legal mother of the child is a woman, who delivers the baby, even though she is not genetically related to the baby. In Europe, a first attempt to specify what surrogate motherhood is at the supranational level, was made by the Ad Hoc* Committee of Experts on Bioethics* (CAHBI), dated January 10, 1989. According to their definition ‘a surrogate mother’ or ‘a surrogate’ is a person who carries a child for another person and who, prior to becoming pregnant, consented to the transfer of the child to that other person after birth [4]. In the literature on the subject a person/couple who concludes a contract with a surrogate mother acting only as a gestational mother or also as an egg donor is called the ‘commissioning party’ [4]. There are two kinds of surrogacy. The first is ‘traditional surrogacy’, ‘straight surrogacy’ or ‘partial surrogacy’, where the surrogate mother uses her own egg and is artificially inseminated using sperm from the intended father or a donor [5]. The second is ‘gestational surrogacy’ or ‘full surrogacy”, where the surrogate mother receives an embryo formed in vitro fertilization (IVF) from the gametes of the ‘intended parents’ or ‘commissioning couple’ so she is not genetically related with the baby she delivers [6].

According to the World Health Organization 15% of couples in their reproductive age and 186 million individuals face infertility problems globally [7]. Over the last years, there have been many advances and improvements in infertility treatments offered to people who desire to have a child. Surrogate motherhood is considered as one of the most controversial methods of infertility treatment associated with difficult ethical, psychological and social issues [8–10]. The literature has shown that individuals’ choices related to surrogacy are grounded by their societal principles including moral, religious and philosophical values and traditions, which often are entwined with ethical and social implications [11]. Hence, all of the different aspects of surrogacy can lead to different trends in attitudes [9]. What is more, globally the legal framework for surrogacy does not exist, thus there are different legal regulations of surrogacy across countries. Countries such as Russia and Ukraine allow commercial and altruistic surrogacy, whereas The United Kingdom, Australia, and Canada legally allow only altruistic surrogacies. In contrast, Germany, France and Italy are among countries, which ban all forms of surrogacy.

Scientific studies across countries examining general opinions about surrogacy have shown conflicting results. An Australian study showed that 75% of respondents presented a positive general attitude towards surrogacy, which was a high increase in comparison to previous research in Australia [1]. A Swedish study among physicians working within obstetrics also indicated that 63% of the sample showed support or were neutral about the concept of surrogacy surrogacy [12]. However, they also were concerned about surrogate mothers’ health and the risk of coercion surrogacy [12]. A review prepared by Rodriguez-Jaume et al. (2021) has shown that a high level of acceptance of surrogacy occurs in countries such as Canada, Japan, the United Kingdom, and Iran. However, in Germany the degree of acceptance of surrogacy is lower. What is interesting, social acceptance of surrogacy was not higher among people with infertility. Six of the eight studies, including infertile groups, showed acceptance rates below the average overall value [13]. Research among the Spanish population has shown that 60.1% of respondents claimed that surrogacy is a “good way to help infertile or homosexual couples to have a child”[13]. In addition, some researchers draw attention that the general opinion about surrogacy can also be influenced by a prevailing negative case portrayal in the media [14].

Some research studies examined attitudes about the controversial aspects of surrogacy. In terms of preference for a genetic bond in surrogacy, the Greek population showed 51.3% of acceptance for traditional surrogacy and 82.1% for gestational surrogacy. The question “If a surrogate mother should receive monetary compensation” also brings controversy. For example, in France commercial surrogacy was considered less problematic than altruistic surrogacy [15]. While altruistic surrogacy is legal in some countries, it is still not clear what is the more favorable practice. In an Australian study 53% of the study group supported altruistic surrogacy [16], where in Canada 24.4% of the population stated approval for commercial surrogacy [17]. The nature of the relationship between a surrogate mother and the commissioning couple can also be complicated. In countries such as the United Kingdom, Turkey and Iran, there is an opinion that surrogate mothers should be a relative of the intended parents, whereases in Greece and Japan the preference is that the surrogate mother should be a stranger [13].

As described above, attitudes towards surrogacy vary from country to country and can be related with socio-demographical factors such as gender [18] age, and socioeconomic status [19]. What is more, positive or negative portrayals of surrogacy in the media [14] and cultural beliefs about parenthood likely shape the opinions of the general population [20]. Nevertheless, the available research results are limited. Despite the discourse about the different aspects of surrogacy in some countries, few researchers answer the question about what the general public attitude on surrogacy is and what exactly determines peoples’ attitudes towards surrogacy [6, 21]. It is very difficult to draw a conclusion from the available scientific studies about attitudes towards surrogacy, as only careful descriptions of some trends are possible. Thus, the aim of this study was develop a tool that would help to examine attitudes toward surrogacy in Poland [13]. The survey in this paper was developed based on the literature and other existing questionaries [9, 10, 12, 22]. Some of the questions were translated and adapted from the previous questionnaires or scales, which are going to be described more in details. Firstly, The Attitude Toward Surrogacy Questionnaire is a German questionnaire, which was developed by Mohnke et al., (2019) [10]. The questionnaire consists of 13 items, enables the examination of public opinion about surrogacy and whether participants could imagine using surrogacy for themselves and others. In the questionnaire there are three factors: general attitude towards surrogacy (9 items), attitude towards monetary compensation (2 items) and attitude towards surrogate mother (2 items). The questionnaire enables an assessment of positive or negative public attitudes towards these three aspects of surrogacy [10]. Second scale, The Attitude Towards Gestational Surrogacy Scale (GSAS) was developed by Rahimi Kian et al., (2016) [9]. It is a comprehensive 30-item tool, which enables the measurement of surrogacy attitudes among infertile couples. GSAS consist of the following 5 subscales: ‘acceptance of surrogacy’, ‘surrogacy and public attitudes’, ‘child born through surrogacy’, ‘surrogate mother’, and ‘intentional attitude’ and ‘surrogacy future attempt’. This scale covers major issues related to gestational surrogacy. The higher the score the more positive the attitude toward surrogacy, and the lower the score the more negative the attitude toward surrogacy. Development of GSAS was tested on 200 infertile couples [9]. Third questionnaire, developed by Swedish scientists was created on the basis of clinical experience and earlier research. The attitude towards surrogacy questionnaire [12] a validated tool, consisted of 38 items, which aimed to assess the opinions and attitudes about surrogacy among physicians. The measure consisted of 3 domains: attitudes towards surrogacy, assessments of prospective surrogate mothers and antenatal and obstetric care for surrogate mothers [12]. Finally, in the study conducted by Rahmani, et al., (2014) viewpoints of fertile women on gestational surrogacy were examined. The scale consisted of 22 items, which were divided into five aspects of gestational surrogacy including legal and religious issues (7 items), conditions for the use of surrogacy (4 items), children born through surrogacy (5 items), the surrogate mother (2 items) and tendency to use surrogacy (4 items) [22].

To sum up, the presented studies aimed to survey opinions about surrogacy. All of the measurements are self-designed, with reliability and validation described above. More detailed comparison between the above tools, including comparison to the scale developed in this paper will be presented in the discussion section. The opinion of the Polish population about surrogacy has not been yet explored. To our knowledge there is no tool available in Polish language to measure the attitudes towards surrogacy with psychometric properties. Thus, constructing a validated scale that allows for an in-depth study of public opinion on surrogacy is needed. In Poland surrogacy is not regulated by law. Thus, it would be important to examine different aspects of surrogacy and check if the opinion towards surrogacy is positive or negative. Researching the attitudes, opinions and societal knowledge about a controversial topic such as surrogacy helps to find out more about potential future behaviours and possible legal solutions in the process of converting controversial topics into normative ones [23]. Open discussion about surrogacy is needed, not only for future regulations, but also helping people make more informed choices about their future by increasing adequate education, awareness and knowledge related to the topic.

Thus, the main aim of this study is to develop a scale for examining the attitudes toward surrogacy in Poland. Secondary aim is to demonstrate validity of the scale through the confirmatory factor analysis. The third aim is to analyze socio-demographical factors contributing to positive or negative attitude to surrogacy parenthood among adult members of the public.

## Materials and methods

### Measure

The study had a cross-sectional design. The development process of the Attitudes towards Surrogacy Scale (ATSS) was based on theoretical and practical knowledge and consisted of a few stages. Firstly, a review of the literature was performed, to find Polish and English questionnaires or scales measuring the attitudes towards surrogacy. The main aspects of surrogacy were selected based on the literature and existing questionnaires: General opinion towards surrogacy, Acceptance of surrogacy, Attitude towards monetary compensation, Attitude towards surrogate mothers, Attitude towards Child born through surrogacy, Surrogacy and public attitudes, Intentional attitude, and surrogacy future attempt. All the questions from validated questionnaires and scales on attitudes towards surrogacy available in the English language [9, 10, 12, 22]. were independently translated into Polish language by 2 qualified translators. Then the relevant item pool was appointed by the Expert Advisory Panel Board, who first commented and judged the items retrieved from the four existing questionnaires. They assessed if the retrieved items were relevant and acceptable in the target population as they are in the original population. Both conceptual and item equivalence was assessed through a literature review. Then the Expert Advisory Panel Board adapted and formulated 24 questions appropriate to the Polish cultural context and grouped them into 4 subscales. A pilot study (N = 30) was conducted to check the comprehensiveness and feasibility (participants were asked about opinions regarding readability, semantics, cultural adaptability. The survey was revised based on the comments. The 24-item version with linguistic adaptation was constructed including 4 aspects of surrogacy:

- general opinion on surrogacy and its social context (7 items),
- financing and legalizing surrogacy (8 items),
- the acceptance of surrogacy (4 items),
- attitudes towards the intended parents and children born through surrogacy (5 items).

The 24-item version was administered to participants in a formal study. The Expert Advisory Panel Board consisted of 8 experienced health care professionals working in the field of obstetrics and reproductive medicine (2 academic who specializes in Health Psychology, 2 specialists who work with infertile couple, 2 gynecologists and 2 obstetricians), who agreed for voluntary expertise in this study.” The survey used in this study is provided as a Supplementary file. The respondents’ opinion was indicated by using a seven-point Likert-type scale (1 = strongly disagree, 7 = strongly agree). For most items a higher score indicates a more positive attitude towards surrogacy. To prevent an acquiescence response bias, 10 items were reversed. Total score was obtained by summing the items, ranging from 15 (minimum negative attitude) to 105 (maximum positive attitude). Prior to the Attitudes Towards Surrogacy survey, socio-demographic data was collected. Socio-demographic questions included: gender, age, place of living, educational background, work background, income, relationship status, sexual orientation, having children, and religion.

### Ethical approval

The study had a cross sectional design and was approved by the Research Ethics Board at the University of Gdansk, Poland (number 46/2020) and abided by the standards of the Declaration of Helsinki.

### Procedure

An online survey was constructed using the Forms website and took approximately 15 min to fill out. The invitations to participate in this study were distributed through various online institutional websites (clinics, hospitals, universities etc.) and other websites such as social platforms (Research Gate; Facebook, infertility groups and forums, parental groups etc.). Participation in the project was voluntary and could be discontinued at any time. Participants were guaranteed that all data was anonymized in its collection, storage, and in the publication of research material. Informed consent to participate in the study has been obtained from all the participants. Prior to filling out the survey, participants were introduced to the definition of surrogacy. All items required a response throughout the whole study – i.e., participants could not complete the questionnaire without leaving an answer to each question.

### Participants

Adult members of the public were recruited. The participants in this study had to meet the following inclusion criteria: (1) no restriction to gender, (2) age ≥ 18, (3) able to complete and understand the questionnaire in Polish, (4) signed informed consent, (5) access to the internet, and (6) permanent residence in Poland. If at least one of the inclusion criteria was not met, the participants were excluded from the study. There was no reward for taking part in this study. It is important to add that 3 participants reported a diagnosed infertility. We decide to not exclude participants with fertility struggles from the study, as literature shows that diagnosis of infertility itself does not change the attitude towards gestational surrogacy [24, 25]. The final sample included 442 individuals (344 female and 78 male) aged from 18 to 72 years old (M = 32.58, SD = 12.96). For a sample composition regarding more specific demographic characteristics see Table 1.

**Table 1:** Sample composition

Variable		N (%) or M (SD)
Gender	Female	344 (78)
	Male	78 (18)
Age		32.58 (12.96)
Place of residence	Town up to 50,000 residents	72 (16)
	City up to 50,000 residents	72 (16)
	City from 50,000 up to 150,000 residents	56 (13)
	City from 150,000 up to 500,000 residents	115 (26)
	City over 500,000 residents	127 (29)
Education	Vocational	10 (2)
	Secondary	212 (48)
	Undergraduate	38 (9)
	Graduate	182 (41)
Occupational status	Employed	235 (53)
	Student	202 (46)
	Unemployed	5 (1)
Link with the medical profession	Yes	244 (55)
	No	198 (45)
Number of people in the household	One	55 (12)
	Two	132 (30)
	Three	106 (24)
	Four	101 (23)
	Five or more	48 (11)
Household income		9,427 (11,429)^a^
Current partnership relations	Single	97 (22)
	Married	146 (33)
	In an informal relationship	177 (40)
	Divorced	12 (3)
	In separation	8 (2)
	Widow/widower	2 (< 1)
Sexual orientation	Heterosexual	398 (90)
	Bisexual	30 (7)
	Homosexual	13 (3)
	Asexual	1 (< 1)
Religion	Catholic	253 (57)
	Other than Catholic	22 (5)
	Atheist	167 (38)
Own child/children	Yes	156 (35)
	No	286 (65)
Have you heard of a surrogate before?	Yes	427 (97)
	No	15 (3)

*N* = 442, ^a^*N* = 329

### Statistical analysis

The statistical analysis consisted of several steps. First, item analysis was performed. Since, we used in the ATSS 7-point Likert scale, we referred to item responses as an ordinal approximation of a continuous variables [26–28]. Moreover, we assessed the normality of item responses to ensure that the calculation of means and standard deviations would be accurate. In line with Curran, West and Finch [29] suggestions, normality was assessed for each of items based on skewness and kurtosis that skewness values > 2 and kurtoses values > 7 were considerate as problematic in the analyses. To assess the responses to individual scale items, the mean, standard deviation, skewness and kurtosis were calculated for each of them. As can be seen in supplementary material Table A1, skewness and kurtosis analysis do not indicate problems with normality.

Then, a series of confirmatory factor analyses (CFAs) was performed. We conducted confirmatory factor analysis to test the initial 4-factor model with the 24 items: (1) general opinion on surrogacy and its social context, 7 items; (2) financing and legalizing surrogacy, 8 items; (3) the acceptance of surrogacy, 4 items; (4) attitudes towards the intended parents and children born through surrogacy, 5 items. For CFA standardized loading coefficients and covariances between factors see supplementary material Table A2. As a result of testing subsequent models (starting with a four-factor model with the original assignment of 24 items), items with factor loadings less than 0.4 were removed [30]. The maximum likelihood with robust standard errors estimator (MLM) was used to evaluate the structure of the scale. In the final model-fit evaluation we relied on the comparative fit index (CFI > 0.90) and root mean square error of approximation (RMSEA < 0.08) [31]. Next, Cronbach’s alpha [32] and McDonald’s omega [33] were used for evaluating the reliability of the finale version of the Attitudes Towards Surrogacy Scale. In internal consistence, a score equal to 0.7 or higher was considered as the acceptable reliability reliability [34]. Finally, multiple linear regression analyses were performed in order to show differences in attitudes towards surrogacy regarding various socio-demographic variables. Statistical analysis was performed using R environment environment [35] along with the appropriate packages: psych [36] and lavaan [37].

## RESULTS

### The Evolution of the ATSS from a 4-factor with 24 Items to a 3-factor with 15 Items

Since the results of the initial confirmatory factor analysis of 4-factor with 24 items model showed poor model fit (Chi-square = 1013.73, *p* < 0.01, df = 246, CFI = 0.83, TLI = 0.82, RMSEA = 0.084 (90% C.I.: 0.079–0.089), SRMR = 0.090), we provided some modifications to improve the model. Apart from the reported statistical information, we also considered the content of the items in the development of final constructs [38, 39]. The list of modifications includes:

1. Removing items with low factor loading: 5, 6, 7, 8 (from *General Opinion on Surrogacy and its Social Context* subscale); 8 (from *Financing and Legalizing Surrogacy* subscale); 17 (from *Acceptance of Surrogacy* subscale), and 20, 22, 23, 24 (from *Attitudes towards the Intended Parents and Children Born through Surrogacy* subscale).
2. Adding item 21 to the first factor and renaming it into *'Surrogacy Ethical Context'*. All but one of the 'Attitudes towards the Intended Parents and Children Born through Surrogacy' factor items have been removed. After careful analysis of their content, it was established that two of them refer to the right of children born through surrogacy to know about the circumstances of their birth. This dilemma probably extends beyond the attitude towards surrogacy itself. In turn, the items removed from the 'General Opinion on Surrogacy and its Social Context' subscale referred to the assessment of how surrogacy is perceived in society, and not by the respondent himself. Finally, reviewing the content of the remaining items from these two subscales led to the decision to combine them under the name 'Surrogacy Ethical Context'.

Next, we conducted confirmatory factor analysis to test the improved 3-factor model with the 15 items: (1) surrogacy ethical context, 4 items; (2) acceptance of surrogacy, 4 items; (3) financing and legalizing surrogacy, 7 items. For CFA standardized loading coefficients and covariances between factors see Table A3 in the supplementary material. The results showed that the three-factor model indicated an acceptable model fit (see *Factor Structure and Reliability* section).

The final version of the ATSS consists of 15 items making up three subscales: (1) Surrogacy Ethical Context, 4 items; (2) Acceptance of Surrogacy, 4 items; (3) Financing and Legalizing Surrogacy, 7 items. For the sake of clarity, in the following subsections the presentation of results has been limited to the data on the final version of the scale, including item analysis.

### Item analysis

To assess the responses to individual scale items, the mean, standard deviation, skewness and kurtosis were calculated for each of them (see Table 2). The inspection of means values showed that individual items were assessed differently by the respondents on average. The opinions regarding the availability of surrogacy for heterosexual couples (*M* = 5.28) and the recognition that surrogacy is a good alternative for people who have already exhausted other possibilities of having a child (*M* = 5.31) were the most widely accepted. Relatively high support was also given to the statement that surrogacy in Poland should be legalized (*M* = 5.08). On the other hand, the lowest rated item by respondents is the decision to surrogate yourself in case of problems with having a baby (*M* = 3.55). A significant proportion of the respondents also admitted that surrogacy raises ethical (*M* = 3.76, reverse coded) and religious (*M* = 3.88, reverse coded) controversies in society. Examination of standard deviations shows that the dispersion of respondents’ scores on items are comparable. A review of skewness and kurtosis did not reveal substantial deviations of the response distributions from the normal distribution.

**Table 2:** Descriptive statistics for the ATSS items

Item [Polish language version]		Subscale	M	SD	Skew	Kurt
1	Surrogacy conflicts with ethical or social principles. [Surogacja jest w konflikcie z zasadami etycznymi lub społecznymi.] (*Reverse coded*)	Surrogacy Ethical Context	4.80	1.70	-0.46	-0.72
2	Surrogacy conflicts with most religious denominations. / Surogacja jest w konflikcie z większością wyznań religijnych. (*Reverse coded*)	Surrogacy Ethical Context	3.88	1.48	0.25	-0.49
3	Surrogacy has serious ethical or social consequences. [Surogacja wiąże się z trudnymi konsekwencjami etycznymi lub społecznymi.] (*Reverse coded*)	Surrogacy Ethical Context	3.76	1.71	0.31	-0.91
4	Children born through surrogacy are at risk of worse mental functioning. [Dzieci urodzone na drodze surogacji są w grupie ryzyka gorszego funkcjonowania psychicznego.] (*Reverse coded*)	Surrogacy Ethical Context	4.95	1.41	-0.25	-0.69
5	My general opinion on surrogacy is positive. [Moja ogólna opinia o surogacji jest pozytywna.]	Acceptance of Surrogacy	4.84	1.61	-0.62	-0.20
6	Surrogacy is a good alternative for people who have already exhausted other possibilities of having a child with their own genetic characteristics (they have undergone many years of expensive therapies. have made unsuccessful attempts at in vitro fertilization). [Surogacja jest dobrą alternatywą dla osób. które wyczerpały już inne możliwości na posiadanie dziecka z własnymi cechami genetycznymi (przeszły wieloletnie. kosztowne terapie. podejmowały nieudane próby zapłodnień in vitro).]	Acceptance of Surrogacy	5.31	1.61	-1.19	0.85
7	Since surrogacy in Poland is not legally regulated, if my friend wanted to conceive a child by surrogacy, I would advise him or her to a surrogacy arrangement abroad. [Ponieważ w Polsce surogacja nie jest uregulowana prawnie, to gdyby mój przyjaciel/moja przyjaciółka chciał/a począć dziecko na drodze surogacji, to doradziłbym/doradziłabym jej/jemu, aby postarać się o surogację za granicą.]	Acceptance of Surrogacy	4.55	1.49	-0.35	-0.24
8	If me or my partner could not conceive a child on our own, I would consider surrogacy. [Jeśli ja lub mój partner/moja partnerka nie moglibyśmy samodzielnie począć dziecka, rozważałabym/rozważyłabym surogację.]	Acceptance of Surrogacy	3.55	1.70	0.18	-0.77
9	Surrogacy in Poland should be legalized. [Surogacja w Polsce powinna być zalegalizowana.]	Financing and Legalizing Surrogacy	5.08	1.62	-0.79	0.04
10	Surrogation should be allowed for infertile heterosexual couples. [Należy zezwolić na surogację dla niepłodnych par heteroseksualnych.]	Financing and Legalizing Surrogacy	5.28	1.63	-0.98	0.27
11	A surrogate for same-sex couples should be allowed. [Należy zezwolić na surogację dla par tej samej płci	Financing and Legalizing Surrogacy	4.26	1.98	-0.21	-1.14
12	People who are single should be allowed to surrogate. [Należy zezwolić na surogację osobom, które są singlami.]	Financing and Legalizing Surrogacy	4.20	1.82	-0.15	-1.00
13	Surrogacy as a method of assisted reproduction should be financed by public funds. [Surogacja jako metoda wspomaganego rozrodu powinna być finansowana przez fundusze publiczne/Narodowy Fundusz Zdrowia.]	Financing and Legalizing Surrogacy	3.89	1.74	0.03	-0.90
14	Paid surrogacy consists of paying the costs related to pregnancy and childbirth and transferring the remuneration to the surrogate mother. I support a commercial surrogate. [Surogacja odpłatna polega na uiszczeniu kosztów związanych z ciążą i porodem oraz przekazaniu wynagrodzenia matce zastępczej. Popieram surogację komercyjną.]	Financing and Legalizing Surrogacy	4.27	1.71	-0.38	-0.70
15	In an altruistic surrogacy, the surrogate mother does not receive any remuneration for giving birth to a child. Future parents pay all costs related to the conception, maintenance, and management of the pregnancy, as well as the delivery itself. I support an altruistic surrogate. [W surogacji altruistycznej matka zastępcza nie otrzymuje żadnego wynagrodzenia za urodzenie dziecka. Przyszli rodzice opłacają wszystkie koszty związane z zapłodnieniem, utrzymaniem i prowadzeniem ciąży a także samym porodem. Popieram surogację altruistyczną.]	Financing and Legalizing Surrogacy	4.30	1.73	-0.28	-0.86

*N* = 442

### Factor structure and reliability

**Fig. 1:**
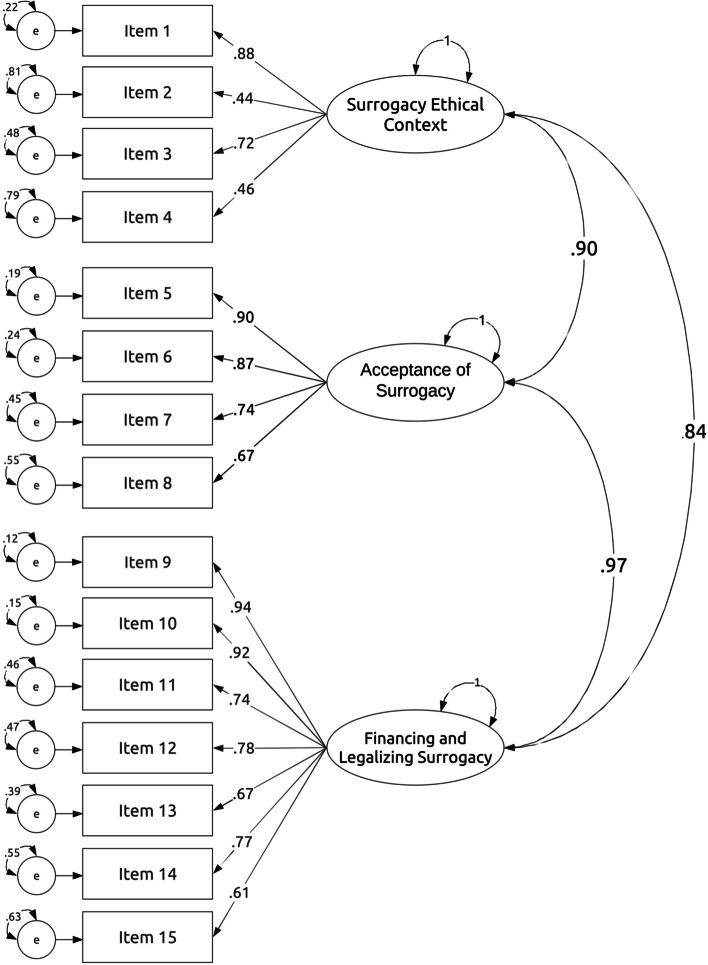
Results of confirmatory factor analysis (CFA). Standardised coefficients are shown

The confirmatory factor analysis (CFA) results for the finale version of the ATSS showed that the three-factor model indicated an acceptable model fit: Chi-square = 320.46, p < 0.01, df = 87, CFI = 0.94, TLI = 0.92, RMSEA = 0.078 (90% C.I.: 0.070–0.086), SRMR = 0.040 (for CFA standardized loading coefficients and covariances between factors see Fig. 1). Reliability was assessed by calculating the McDonald's omega for each of the three subscales, as well as for overall ATSS score. The omega coefficient for the Surrogacy Ethical Context subscale was 0.74, for the Acceptance of Surrogacy subscale was 0.88, for the Financing and Legalizing Surrogacy subscale was 0.92, and for the General Attitude towards Surrogacy score was 0.94. Additionally, bearing in mind that ATSS results are generated by calculating a mean response across a set of questions (each item is equally included in the calculation of a score), internal consistency was also assessed by calculating the Cronbach's alpha as tau-equivalent reliability (under assumption that all the items in the scale have the same relationship with the underlying construct). So, the alpha coefficients for the three subscales and overall ATSS score were respectively: 0.73, 0.88, 0.91, and 0.94.

### Attitude towards surrogacy and sociodemographic variables

To model the linear relationship between selected sociodemographic variables and attitudes towards surrogacy, multiple linear regression analyses were used. The following variables were considered as hypothetical predictors of attitudes towards surrogacy: age, gender, education, study, link with the medical profession, type of relationship, having a child and being a religious believer. The analysis included variables for which complete data was collected. Some categorical variables have been aggregated to ensure the appropriate size in each category (minimum 20% of the sample). Thus, the education categories included: vocational or secondary versus undergraduate or graduate education; type of relationship categories included: being married versus being in an informal relationship versus being single, divorced or in separation; religion categories included: being a religious believer versus being atheist. To assess simultaneously the effect of several predictors on various facets of attitude towards surrogacy the analysis was conducted with adjustment for all sociodemographic factors. Table 3 shows the results of multiple regression for the three facets of attitude towards surrogacy as well as for the overall score.

**Table 3:** Results of a multiple linear regression analyses (unstandardized coefficients) for demographic variables predicting attitudes towards surrogacy

Predictor	Surrogacy Ethical Context		Acceptance of Surrogacy		Financing and Legalizing Surrogacy		General Attitude toward Surrogacy	
	B	SE	B	SE	B	SE	B	SE
*Intercept*	4.73	.28	5.08	.31	4.93	.31	4.94	.28
Age	.01	.28	.01	.01	.02	.01	.01	.01
Gender: male	-.10	.15	-.15	.17	.07	.16	-.08	.15
Vocational or secondary versus undergraduate or graduate education	.38*	.15	.28	.18	.32	.17	.31	.16
Study	-.27	.21	-.20	.24	-.19	.24	-.22	.21
Link with the medical profession	-.27*	.12	-.26	.14	-.33*	.14	-.28*	.13
Being married versus in an informal relationship	-.17	.18	-.41*	.20	-.46*	.20	-.36*	.18
Being single versus in an informal relationship	.03	.14	-.23	.16	-.23	.16	-.15	.14
Own child/children	-.05	.19	-.48*	.21	-.27	.21	-.31	.19
Being a religious believer versus atheist	-.45***	.12	-.95***	.14	-.72***	.14	-.75***	.12
**Adjusted R**^**2**^	.05***		.15***		.10***		.12***	

*N* = 442, * *p* < 0.05, ** *p* < 0.01, *** *p* < 0.001

As can be seen in Table 3, age, gender, continuing to study, nor being single were not significant predictors of any aspect of attitude towards surrogacy. It was found that the most significant predictor of all three aspects of the attitude towards surrogacy, as well as the general attitude towards it, was being a believer (Catholic or a follower of another religion).

## Discussion

### Attitude towards Surrogacy Scale and comparison with other surrogacy measures

Surrogacy is not regulated by law in Poland, however, it is present in the society. Thus, the aim of the study was to develop a scale with psychometric properties, which enables the measurement of the general attitude towards surrogacy among the Polish population. The present scale has a total of 15 items that allows to capture the opinions towards three aspects of surrogacy: surrogacy’s ethical context, financing and legalizing surrogacy, and acceptance of surrogacy. To our knowledge this is the first scale assessing attitudes towards.

Studies conducted around the world, which measured attitude towards surrogacy, mostly used self-designed measures. However, the validation process and used methods often are not fully explained. In this study, the developed scale has Cronbach’s alpha and omega coefficient were used as reliability measurements. Both measurements show satisfactory reliability of this scale, which also is comparable to studies conducted by Rahimi Kian et al., (2016); Mohnke et al., 2019; and Rahmani et al., (2014). In contrast Poote and van den Akker (2009) did not apply Cronbach’s alpha or other validity and reliability measurements [40]. Similarly to other studies, this paper used the Likert Scale for scoring items [9, 10, 22, 40]. The statistical approach in this study is different than in other research. In this study for validation purpose, a series of Confirmatory Factor Analyses (CFAs) was performed on a pool of items to identify essential items and group them into factors. Where in comparison Mohnke et al., (2019) and Chliaoutakis et al., (2002) conducted a Principal Component Analysis (PCA) [10, 41]. Rahimi Kian, et al. (2016) applied a different approach, in which qualitative and quantitative content validity approaches were applied in the pilot study (*N* = 30) and the data pool was enhanced by the comments provided by the members of an Expert Advisory Panel [9]. In some previous research, an Expert Advisory Panel and a pilot study were not included in the process of scale development [21]. While some other research includes an Expert Advisory Panel pilot study [9, 12] or only just a pilot study [10, 22].

The range of surrogacy factors classified in this study is comparable to other scales and questionnaires [9, 10, 22]. For example, in the German Attitude Towards Surrogacy Questionnaire developed by Mohnke et al., (2019) attitude towards monetary compensation included only two items, related to altruistic and commercial surrogacy [10]. However, in our scale the financial and legal context of surrogacy was condensed into one factor – financial and legal context. Additionally, our scale has one question regarding if surrogacy should be legalized and one question if surrogacy should be publicly founded, which were not included in the German Scale. In terms of the legal aspect of surrogacy there are 7 items in this scale, which allows the assessment of opinions about legalizing surrogacy among not only heterosexual couples, but also same-sex couples and singles. Similarly, no detailed questions like the above are included in the Scale developed by Rahmani et al., (2014) or Rahimi Kian et al., (2016). Many advances in assisted reproduction technology are facilitating the development of new types of families. Recently, researchers have shown that the concept of family is changing, and new types of families are emerging, including same-sex parenting. Thus, including items related to the attitude towards surrogacy for other groups other than heterosexual is important [42]. The items related to the acceptance of surrogacy in our scale consisted of 4 items, which are similar to those in the German questionnaire [10]. However, the German questionnaire consists of 9 items, and these are included in the general attitude towards surrogacy aspect. The ethical context of surrogacy in this paper consists of 4 items, which covered religion, if surrogacy conflicts with ethical and social rules, and the negative social and ethical consequences of surrogacy. Some of the items related to the acceptance of surrogacy are like those in the scale developed by Rahmani et al., (2014) or Rahimi Kian et al., (2016).

From 24 items in the original survey used in this study, 9 items dropped out based on the conducted statistical analysis. Obtained results showed that those items in this study do not differentiate participants who have positive or negative attitudes towards surrogacy. It can be assumed that those items are more related to the general opinion about the current political and social context in Poland, which also can be related with the participants answers.

### Attitude towards surrogacy in Poland

The respondents in this study declared the highest support for surrogacy as a good option for heterosexual couples (in comparison with same sex couples or singles) and those who have tried all other alternatives to have a baby. Similarly, a Romanian study also demonstrated opinion that surrogacy should be available only for heterosexual couples [43]. In contrast, surrogacy as an option to have a baby for same sex couples was acceptable among Swedish physicians [12, 44]. Swedish physicians were also less supportive for the surrogacy among singles [12].

The lowest support in this study sample was related to the statement “If me or my partner could not conceive a child on our own, I would consider surrogacy”. Research conducted by Chliaoutakis et al., (2002) showed that the public attitude towards a willingness for using gamete donation and surrogacy or encourage a family member to do the same, is divided, where specifically, the majority of participants had a negative attitude about using surrogacy [41]. In the study conducted by (Rahmani et al., 2014) among Muslim women a significant percentage of the sample would not recommend surrogacy to women with infertility and claimed that.adoption would be a better solution than surrogacy [22]. Researchers have presented that the main motivation of intended parents to use gestational surrogacy as a method to have a child is based on the desire to have a genetic connection with their baby in comparison with adoption [45]. Intended parents want to be able to participate in the child’s life and development process from the very beginning [42].

Obtained findings in this study, showed that a significant proportion of the respondents believed that surrogacy raises ethical and religious controversies in the society. People very often perceive new innovations or technologies through social and cultural norms, values, and traditions, which are normative in the population [21]. The findings are in line with other research, where religious beliefs play an important role in shaping opinions about surrogacy and also assisted reproductive methods [22, 46].

The literature on the subject has shown that individuals’ choices related to surrogacy are grounded by their societal principles including moral, religious and philosophical values and traditions, which are often entwined with ethical and social implications [47]. Surrogate parenthood can be further complicated by ethical and moral dilemmas when some intended couples looking for a surrogate may offer financial compensation [48]. Studies has shown that altruism is the main motivation for being involved in surrogacy [21]. Thus, in some countries such as the United Kingdom, Belgium or Netherlands altruistic surrogacy is legal [21, 46, 49]. Currently, Poland has no law regulating surrogacy, which creates legal ambiguities. Respondents in this study declared high support for surrogacy to be legalized.

Current trends towards surrogacy are different in different countries. Surrogacy is related with complex aspects, thus is considered as the most controversial method of all the reproductive options. A general positive attitude towards surrogacy does necessarily mean that a couple would pursue the surrogacy option themselves. Some aspects of surrogacy (for example commercial surrogacy) can be related with negative attitudes and lack of acceptance. Thus, it is important to research attitudes towards surrogacy including its various aspects.

### Attitudes towards surrogacy in Poland and socio-demographic factors

The analysis of attitude towards surrogacy with socio-demographic variables showed that age, gender, level of education, or being single were not significant predictors of any aspect of attitude towards surrogacy. In this study the most significant predictor of the general attitude towards surrogacy, and three aspects of surrogacy was being a religious person (profess a Catholic religion or profess another religion). Religious respondents indicated ethical and social objections to surrogacy, were less supportive of financing and legalizing surrogacy, and were more skeptical about surrogacy as one of the options in reproductive medicine. Similarly, in other conducted studies religious respondents are less willing to support and be positive about surrogacy [41]. It can be claimed that religion and religious beliefs play a significant role in shaping attitudes towards surrogacy. In our study the majority of the participants (57%) declared being a Catholic or believing in another religion, while 38% declared themselves as atheist. Religions, which exist around the world present different approaches to surrogacy [46]. According to the Catholic stance, the intrusion of a third person into a couple’s relations is immoral and as such they are strongly opposed to surrogacy [46]. Thus, it is not surprising that in Poland, where Catholicism is still a dominant religion, surrogacy and different aspects of surrogacy are often perceived negatively.

Contrary to expectations, the medical profession, being married (versus an informal relationship) or having a child was negatively connected to some aspects of the attitude towards surrogacy. Those participants, who were related with the medical profession, had stronger ethical concerns about surrogacy and had more negative attitudes about the funding and legalization of this method. In this study 55% of participants worked in the health care profession. Swedish research, which included a sample of physicians working in an infertility clinic declared more supportive attitudes towards the legalization and public financing of surrogacy in comparison with those working within antenatal and delivery care. The risk that the commissioning couple might pay the surrogate mother “under the table” was declared by 82% of respondents [12].

Findings in this paper also show that married people, as well as those with children, presented less acceptance of a surrogacy arrangement for those who struggle with reproductive problems. It is a surprising result, since 65% of participants in this study did not have children. Other findings in a review conducted by Rodriguez-Jaume et al., (2021) suggest that social acceptance of surrogacy and positive attitude was not higher among people with infertility [13]. Six of the eight studies including infertile groups showed acceptance rates below the average overall value [13]. In an Australian study, where demographic variables were measured, none of those variables were predictors of attitudes towards surrogacy. Age, education level, or having one’s own children did not correlate with attitudes towards surrogacy [1]. This is in line with the findings in this study.

### Strength of the study

The presented scale measures attitudes towards surrogacy in Poland, which to our knowledge is novel in Poland. The major strength of this study is constructing the scale with the established validity and reliability. There are other research where surrogacy attitude surveys have been developed. However, in many of these the reliability and/or the validation process is not clearly and fully described. Secondly, in the process of scale development a pilot testing was conducted to decrease the risk of bias. Pilot assessments are needed for the scale feasibility, readability of included items and assessment whether they are subjectively perceived by respondents as addressing what they are designed to measure. The other strength of the developed scale is that it can be addressed to different groups and is not only limited to people with infertility.

Assessing the opinions and attitudes on a controversial topic such as surrogacy, plays an important role in disclosing various aspects of surrogacy, helps to fill in legislative gaps and ambiguities, and to convert controversial dimensions surrounding surrogacy into a normative concept that eliminates stigma [9]. We hope that creating this scale will add to the growing body of the literature through the addition of a new tool in reproductive medicine.

### Limitations

To make appropriate interpretation and use of the study findings the limitations need to be acknowledged. The major limitation of this study is a limited external generalizability in terms of a non-representative sample which is linked with the on-line application of the ATSS and low sample size. In terms of representativeness of the sample the majority of the study participants were women 78% (344 female and 78 male), which demonstrates that the male perspective is underrepresented. On the other hand, a higher rate of female participation in this type of research, related to reproduction, is not surprising. Additionally, more than half of the participants were Catholic 57%, and only 38% were atheist, where also the atheist perspective is underrepresented. It is therefore possible, that much of the current sample is a more conservative cross-section of society. The obtained findings should be interpreted with caution, as the scale require further validation studies including broader sample.

### Clinical implications

This paper has important implications by raising the importance of discourse about surrogacy in Poland. To our knowledge, this is the first validated scale, which allows the assessment of attitudes towards surrogacy in Poland. This measure allows to capture the opinions towards three aspects of surrogacy: surrogacy’s ethical context, the financing and legalizing surrogacy, and acceptance of surrogacy. The scale can be addressed to various groups in the population, not only for studies related to reproductive medicine.

### Future research

Further research studies are needed to determine if the developed instrument can accurately measure attitudes towards surrogacy among a broader cross section of the community and with different cultural backgrounds. What is more, variety among participants and their socio-demographic characteristic is needed. In future studies it would be important to expand the knowledge of what exactly shapes the attitude and opinion about surrogacy (factors and variables).

What is more, the diversity of interest in different aspects of surrogacy limits the discussion, as researchers concentrate on different elements of surrogacy. In the literature mostly public opinions and the debate about surrogacy is reviewed in the context of infertility problems, where surrogacy can be an alternative for a biological impossibility. In addition, there is a need to expand the context to different family forms, other than just traditional families. Researching the attitude towards surrogacy should include non-heterosexual and non-heteronormative groups, so they are included in the social debate about surrogacy and in the construction of surrogacy as a social issue regulated by law [13].

## Conclusion

ATSS was developed to measure general attitude toward surrogacy. The ATSS indicates the acceptable psychometric properties. The analysis of socio-demographic variables with ATSS showed that the most significant predictor of the general attitude towards surrogacy, and three aspects of surrogacy was being a religious person (profess a Catholic religion or profess another religion). Surrogacy as a topic has not been researched in Poland. This research creates an opportunity to open a public debate about surrogacy, not only restricted to international incidents of surrogacy presented in media or talking judgmentally about people who travel to other countries to have a child where surrogacy is legal. Research on controversial topics such as surrogacy can contribute to expanding public knowledge about surrogacy, including the different aspects of surrogate parenthood.

## Supplementary Information

**Additional file 1:** **Table A1. **Descriptive statistics, skewness and kurtosis for the ATSS initial versionitems. **Table A2.** CFA standardized loading coefficients and covariancesbetween factors - initial 4-factor model with the 24 items.** Table A3.** CFA standardized loading coefficients andcovariances between factors - finale 3-factor modelwith the 15 items.

## Data Availability

The datasets used during the current study are available from the corresponding author on reasonable request.

## Abbreviations

ATSS: Attitude towards Surrogacy Scale
CFA: Confirmatory Factor Analysis
IVF: In vitro fertilization
CAHBI: Ad Hoc Committee of Experts on Bioethics
GSAS: The Attitude Towards Gestational Surrogacy Scale
