# Supplementary material for: Development and validation of the attitude towards Surrogacy Scale in a polish sample
Source: BMC Pregnancy Childbirth. 2023 Jun 3;23:413. doi: 10.1186/s12884-023-05751-x (PMC10239602; doi:10.1186/s12884-023-05751-x)
Supplement: Supplementary file 1 — Additional file 1: Table A1. Descriptive statistics, skewness and kurtosis for the ATSS initial versionitems. Table A2. CFA standardized loading coefficients and covariancesbetween factors - initial 4-factor model with the 24 items. Table A3. CFA standardized loading coefficients andcovariances between factors - finale 3-factor modelwith the 15 items. [file 12884_2023_5751_MOESM1_ESM.docx]

***Supplementary Materials***

**Table A1.** Descriptive statistics, skewness and kurtosis for the ATSS initial version items.

| **Item** | | **Initial subscale** | **Final subscale** | **M** | **SD** | **Skew.** | **Kurt.** |
| --- | --- | --- | --- | --- | --- | --- | --- |
| 1/5 | My general opinion on surrogacy is positive. | General opinion on surrogacy and its social context | Acceptance of Surrogacy | 4.84 | 1.61 | -0.62 | -0.20 |
| 2/1 | Surrogacy conflicts with ethical or social principles. (*Reverse coded*) | General opinion on surrogacy and its social context | Surrogacy Ethical Context | 4.80 | 1.70 | -0.46 | -0.72 |
| 3/2 | Surrogacy conflicts with most religious denominations. (*Reverse coded*) | General opinion on surrogacy and its social context | Surrogacy Ethical Context | 3.88 | 1.48 | 0.25 | -0.49 |
| 4/3 | Surrogacy has serious ethical or social consequences. (*Reverse coded*) | General opinion on surrogacy and its social context | Surrogacy Ethical Context | 3.76 | 1.71 | 0.31 | -0.91 |
| 5 | Mainly most traditional societies have negative attitudes toward surrogacy. | General opinion on surrogacy and its social context | – | 2.89 | 1.16 | 0.67 | 0.84 |
| 6 | Surrogacy in mass-media is presented surrogacy negatively. | General opinion on surrogacy and its social context | – | 3.46 | 1.30 | 0.23 | -0.44 |
| 7 | Public awareness about surrogacy in a Polish society is low. | General opinion on surrogacy and its social context | – | 2.13 | 1.05 | 1.28 | 2.40 |
| 8 | I believe that there is a need for legal regulation of surrogacy. | Financing and Legalizing Surrogacy | – | 5.52 | 1.31 | -1.17 | 1.46 |
| 9/9 | Surrogacy in Poland should be legalized. | Financing and Legalizing Surrogacy | Financing and Legalizing Surrogacy | 5.08 | 1.62 | -0.79 | 0.04 |
| 10/10 | Surrogation should be allowed for infertile heterosexual couples. | Financing and Legalizing Surrogacy | Financing and Legalizing Surrogacy | 5.28 | 1.63 | -0.98 | 0.27 |
| 11/11 | A surrogate for gay couples should be allowed. | Financing and Legalizing Surrogacy | Financing and Legalizing Surrogacy | 4.26 | 1.98 | -0.21 | -1.14 |
| 12/12 | People who are single should be allowed to surrogate. | Financing and Legalizing Surrogacy | Financing and Legalizing Surrogacy | 4.20 | 1.82 | -0.15 | -1.00 |
| 13/13 | Surrogacy as a method of assisted reproduction should be financed by public funds. | Financing and Legalizing Surrogacy | Financing and Legalizing Surrogacy | 3.89 | 1.74 | 0.03 | -0.90 |
| 14/14 | Paid surrogacy consists in paying the costs related to pregnancy and childbirth and transferring the remuneration to the surrogate mother. I support a commercial surrogate. | Financing and Legalizing Surrogacy | Financing and Legalizing Surrogacy | 4.27 | 1.71 | -0.38 | -0.70 |
| 15/15 | In an altruistic surrogacy, the surrogate mother does not receive any remuneration for giving birth to a child. Future parents pay all costs related to the conception, maintenance and management of the pregnancy, as well as the delivery itself. I support an altruistic surrogate. | Financing and Legalizing Surrogacy | Financing and Legalizing Surrogacy | 4.30 | 1.73 | -0.28 | -0.86 |
| 16/6 | Surrogacy is a good alternative for people who have already exhausted other possibilities of having a child with their own genetic characteristics (they have undergone many years of expensive therapies. have made unsuccessful attempts at in vitro fertilization). | Acceptance of Surrogacy | Acceptance of Surrogacy | 5.31 | 1.61 | -1.19 | 0.85 |
| 17 | I believe that adoption is a more acceptable form of surrogacy than surrogacy. (Reverse coded) | Acceptance of Surrogacy | – | 1.88 | 0.99 | 1.59 | 3.69 |
| 18/7 | Since surrogacy in Poland is not legally regulated, if my friend wanted to conceive a child by surrogacy, I would advise him or her to a surrogacy arrangement abroad. | Acceptance of Surrogacy | Acceptance of Surrogacy | 4.55 | 1.49 | -0.35 | -0.24 |
| 19/8 | If me or my partner could not conceive a child on our own, I would consider surrogacy. | Acceptance of Surrogacy | Acceptance of Surrogacy | 3.55 | 1.70 | 0.18 | -0.77 |
| 20 | Babies born through surrogacy are just as healthy as other babies. | Attitudes towards the intended parents and children born through surrogacy | – | 6.06 | 1.03 | -1.31 | 2.28 |
| 21/4 | Children born through surrogacy are at risk of worse mental functioning. (*Reverse coded*) | Attitudes towards the intended parents and children born through surrogacy | Surrogacy Ethical Context | 4.95 | 1.41 | -0.25 | -0.69 |
| 22 | Children born through surrogacy are at risk of social stigma. (*Reverse coded*) | Attitudes towards the intended parents and children born through surrogacy | – | 3.83 | 1.41 | 0.27 | -0.41 |
| 23 | The child should be informed if it was conceived by surrogacy. | Attitudes towards the intended parents and children born through surrogacy | – | 477 | 1.30 | -0.36 | 0.10 |
| 24 | Children born through surrogacy should know the identity of their surrogate mother. | Attitudes towards the intended parents and children born through surrogacy | – | 3.88 | 1.37 | -0.05 | -0.13 |

*N* = 422

**Table A2.** CFA standardized loading coefficients and covariances between factors - initial 4-factor model with the 24 items.

| **Item** | **General Opinion on Surrogacy and its Social Context** | **Financing and Legalizing Surrogacy** | **Acceptance of Surrogacy** | **Attitudes towards the Intended Parents and Children Born through Surrogacy** |
| --- | --- | --- | --- | --- |
| 2 (r) | 0.88 |  |  |  |
| 3 (r) | 0.43 |  |  |  |
| 4 (r) | 0.71 |  |  |  |
| 5 (r) | 0.10 |  |  |  |
| 6 (r) | –0.15 |  |  |  |
| 7 (r) | ­–0.32 |  |  |  |
| 8 |  | 0.53 |  |  |
| 9 |  | 0.94 |  |  |
| 10 |  | 0.92 |  |  |
| 11 |  | 0.74 |  |  |
| 12 |  | 0.78 |  |  |
| 13 |  | 0.67 |  |  |
| 14 |  | 0.77 |  |  |
| 15 |  | 0.61 |  |  |
| 1 |  |  | 0.90 |  |
| 16 |  |  | 0.87 |  |
| 17 (r) |  |  | 0.06 |  |
| 18 |  |  | 0.74 |  |
| 19 |  |  | 0.67 |  |
| 20 |  |  |  | 0.40 |
| 21 (r) |  |  |  | 0.95 |
| 22 (r) |  |  |  | 0.32 |
| 23 |  |  |  | –0.13 |
| 24 |  |  |  | –0.22 |
| *Covariances* |  |  |  |  |
| **General Opinion on Surrogacy and its Social Context** | | 0.85 | 0.91 | 0.48 |
| **Financing and Legalizing Surrogacy** | |  | 0.97 | 0.46 |
| **Acceptance of Surrogacy** | |  |  | 0.46 |

**Table A3.** CFA standardized loading coefficients and covariances between factors - finale 3-factor model with the 15 items.

| **Item** | **Surrogacy Ethical Context** | | **Acceptance of Surrogacy** | | **Financing and Legalizing Surrogacy** |
| --- | --- | --- | --- | --- | --- |
| 2 (r) | 0.88 | |  | |  |
| 3 (r) | 0.44 | |  | |  |
| 4 (r) | 0.72 | |  | |  |
| 21 (r) | 0.46 | |  | |  |
| 9 |  | | 0.94 | |  |
| 10 |  | | 0.92 | |  |
| 11 |  | | 0.74 | |  |
| 12 |  | | 0.78 | |  |
| 13 |  | | 0.67 | |  |
| 14 |  | | 0.77 | |  |
| 15 |  | | 0.61 | |  |
| 1 |  | |  | | 0.90 |
| 16 |  | |  | | 0.87 |
| 18 |  | |  | | 0.74 |
| 19 |  | |  | | 0.67 |
| *Covariances* |  | |  | |  |
| **Surrogacy Ethical Context** | | 0.90 | | 0.84 | |
| **Acceptance of Surrogacy** | |  | | 0.97 | |
